# Living With Parents-In-Law Increased the Risk of Postpartum Depression in Chinese Women

**DOI:** 10.3389/fpsyt.2021.736306

**Published:** 2021-12-20

**Authors:** Songxu Peng, Xin Lai, Jun Qiu, Yukai Du, Jing Yang, Ying Bai, Yanhong Jia, Liping Meng, Kewei Wang, Xiangyang Zhang

**Affiliations:** ^1^Department of Maternal and Child Health, Xiangya School of Public Health, Central South University, Changsha, China; ^2^Department of Children's Intensive Research Center, Hunan Children's Hospital, Changsha, China; ^3^Department of Maternal and Child Health, School of Public Health, Tongji Medical College, Huazhong University of Science and Technology, Wuhan, China; ^4^Department of Public Health, Baoan Maternal and Child Health Hospital, Jinan University, Shenzhen, China; ^5^CAS Key Laboratory of Mental Health, Institute of Psychology, Chinese Academy of Sciences, Beijing, China

**Keywords:** postpartum depression, living arrangements, interaction, parents-in-law, risk

## Abstract

**Background:** A variety of psychological and socioeconomic factors contribute to the development of postpartum depression (PPD). However, the relationship between maternal living arrangements and PPD is unclear.

**Objective:** To assess the relationship between maternal living arrangements and PPD in Chinese population.

**Methods:** A cross-sectional survey was conducted among puerperal women delivered in Baoan Maternal and Child Health Hospital in Shenzhen, China. The Edinburgh Postnatal Depression Scale (EPDS) was used to assess PPD. A score of ≥10 was used as the threshold for postpartum depression.

**Results:** A total of 4,813 women were recruited, of whom 2,535 (52.7%) lived only with their husbands, 664 (13.8%) lived with their parents, and 1,614 (33.5%) lived with their parents-in-law. Compared with women who lived with husbands, puerperal women who lived with their parents-in-law were more likely to be positive for PPD screening (14.1 vs. 10.5%, *P* < 0.001). After adjusting for other influencing factors, living with parents-in-law was significantly associated with the risk of PPD (OR = 1.38, 95% CI, 1.12–1.70). Additionally, stratification analyses showed that the association between living with parents-in-law and the presence of PPD was more significant in women with anxiety during pregnancy (P for interaction <0.05).

**Conclusions and Relevance:** Our data confirms that the maternal living arrangements affect the risk of PPD, especially among women with anxiety during pregnancy. Therefore, more targeted preventive measures should be taken for postpartum depression in women who live with their parents-in-law.

## Introduction

Postpartum depression (PPD) is a common perinatal complication, affecting 0.5–60% of women worldwide (1, 2). A recent systematic review reported that pooled prevalence of PPD in China was 15% (3). There is accumulating evidence that PPD increases the risk of death for mothers and children (4). Mothers with PPD tend to have a poor marital relationship and impaired social function (5, 6). Previous studies have shown that maternal PPD can impair the cognitive, emotional, and physical development of offspring in infancy, childhood and even adolescence (7–9). Findings from published studies have identified several risk factors associated with PPD, including lack of social support, adverse childhood experiences, stressful life events, unsatisfactory marital relationship, and fetal or neonatal health problems (10–14). It has been hypothesized that there may be underlying biological mechanisms behind these associations.

Personal living arrangements are viewed as an important factor affecting health (15–17). However, previous studies mainly focused on the health problems of the elderly (18, 19). Among older adults, living together is a protective factor for physical and mental health due to daily care and emotional support, which may have the same effect in maternal groups. But few studies have focused with interest on the relationship between living arrangements and the health of puerperal women. In China, multi-generation families used to be the main living arrangement, and puerperal women were usually looked after by their parents-in-law (20). As a result, puerperal women may obtain more family support, which may buffer the risk of PPD. In Chinese traditional culture, offspring are expected to obey their parents, and daughter-in-law are even expected to be submissive in a shared living environment (21–23). However, with economic development and alterations in social factors including the increase in the level of education and the spread of scientific knowledge of parenting, significant changes have taken place among women in the puerperium (24). Hence, living together can easily lead to family conflicts and adversely affect maternal mental health due to the differences in thinking and parenting concepts between older parents and younger mothers. It is therefore a key question as to whether such living arrangements may exacerbate or buffer the risk of PPD in these puerperal women.

A preliminary investigation by Honjo et al. (25) used a national cohort study database to assess the association between co-resident family members and PPD risk and ultimately reported a positive association between living with parents-in-law and PPD risk. However, this study failed to adjust for prenatal emotional factors known to influence PPD, including depression and anxiety during pregnancy. Another cross-sectional study from China found that living with in-laws was associated with an increased risk of PPD (20). However, this study did not explore whether this association was modified by other factors. Furthermore, a Pakistani study yielded inconsistent results with a reduced risk of PPD among women in the puerperium who lived with their in-laws (26). Therefore, this association between maternal living arrangements and PPD remains to be verified.

As China's two-child policy opens up and more mothers begin to have children, they may face cohabitation between mothers-in-law and daughters-in-law. Hence, understanding this relationship is essential for targeting interventions that will reach the most vulnerable in China and other Asian countries where most puerperal women live in multi-generational families. Therefore, we performed this cross-sectional survey to investigate the relationship between maternal living arrangements and the risk of PPD among Chinese women, and explore whether this association was modified by other factors.

## Methods

### Study Design and Participants

This cross-sectional survey was conducted at Baoan Maternity and Child Health Hospital in Shenzhen, China from January 1, 2016 to December 31, 2016. The Edinburgh Postpartum Depression Scale (EPDS) was used to screen all women who gave birth in this hospital and returned to the hospital for routine follow-up 6 weeks after delivery. A total of 5,756 women were invited to participate in this study. Of them, 5,043 (87.6%) women agreed to fill out the questionnaire through face-to-face interviews. Subsequently, 230 participants were excluded due to incomplete data on living conditions or PPD. Finally, this study included 4,813 participants.

The study protocol was approved by the Institutional Review Committee of Baoan Maternity and Child Health Hospital, Jinan University. The methodology followed the principles of the Declaration of Helsinki. All subjects provided written informed consent forms for participation.

### Measures

#### Maternal Living Arrangement

Information about the maternal living arrangements was obtained by asking the following question: Who did you live with after childbirth? In the current study, the living arrangements were divided into three categories: living only with husband, living with parents, and living with parents-in-law.

#### Screening for Postpartum Depression

The Edinburgh Postpartum Depression Scale (EPDS) was used to assess postpartum depression. EPDS consists of 10 items, each with a score of 0–3. The total score ranges from 0 to 30, the higher the score, the greater the risk of PPD. The EPDS has been translated and validated in many countries, with various cut-off points during the postnatal period, such as Turkey (12.5) (27), Malta (11.5) (28), Spain (10.5) (29), and India (8.5) (30). In 1998, Lee et al. translated and validated the Chinese version of the EPDS and identified 9/10 as the optimal cut-off for the study population. Using this cut-off value, the sensitivity of the scale was 82% and the specificity was 86% (31). In this study, a score ≥10 was considered an indicator of PPD, which was consistent with previous study (32). The Cronbach's alpha value of this study was 0.826.

#### Assessment of Covariates

A self-designed questionnaire was used to collect sociodemographic data such as age, education level, employment status, and reproductive history, as well as psychiatric history. Also, we collected pregnancy-related data including gestational diabetes mellitus (GDM), depression and anxiety during pregnancy, pregnancy-induced hypertension (PIH), stressful life events, mode of delivery, gestational age, birth weight, fetal sex, malformation, and feeding pattern. We then checked the accuracy of these data based on the participants' medical records.

#### Statistical Analysis

The continuous variables were expressed as means ± standard deviations (SDs), and the categorical data was present as numbers and proportion (%). Analysis of variance (ANOVA) was used to examine the differences between continuous variables, and chi-squared tests were used to compare the categorical data. PPD was considered a dependent variable (dummy code, 0 = no, 1 = yes), and several logistic regression models were used to estimate the odds ratios (ORs) with 95% confidence intervals (95% CIs) between maternal living arrangements and PPD risk. Subgroup analyses were performed stratified by age (<35, ≥35 years), education level (junior or below, senior middle school, college, or university), employment status (full-time, self-employed, housewife, other), primipara (yes, no), GDM (yes, no), depression during pregnancy (yes, no), anxiety during pregnancy (yes, no), stressful life events (yes, no), male infant (yes, no), cesarean delivery (yes, no), preterm birth (yes, no), low birth weight (yes, no), and feeding pattern at 6 weeks (exclusive, partial, formula only). The multiplicative interaction was tested by a likelihood ratio test in logistic regression model to assess the interaction between other variables and living arrangements. The statistical significance was evaluated at the level of 5% (two-tailed test). Statistical analyses were performed using SPSS 18.0 software (SPSS, Chicago, IL, USA).

## Results

### Sociodemographic and Pregnancy-Related Characteristics of Participants

**Figure 1:**
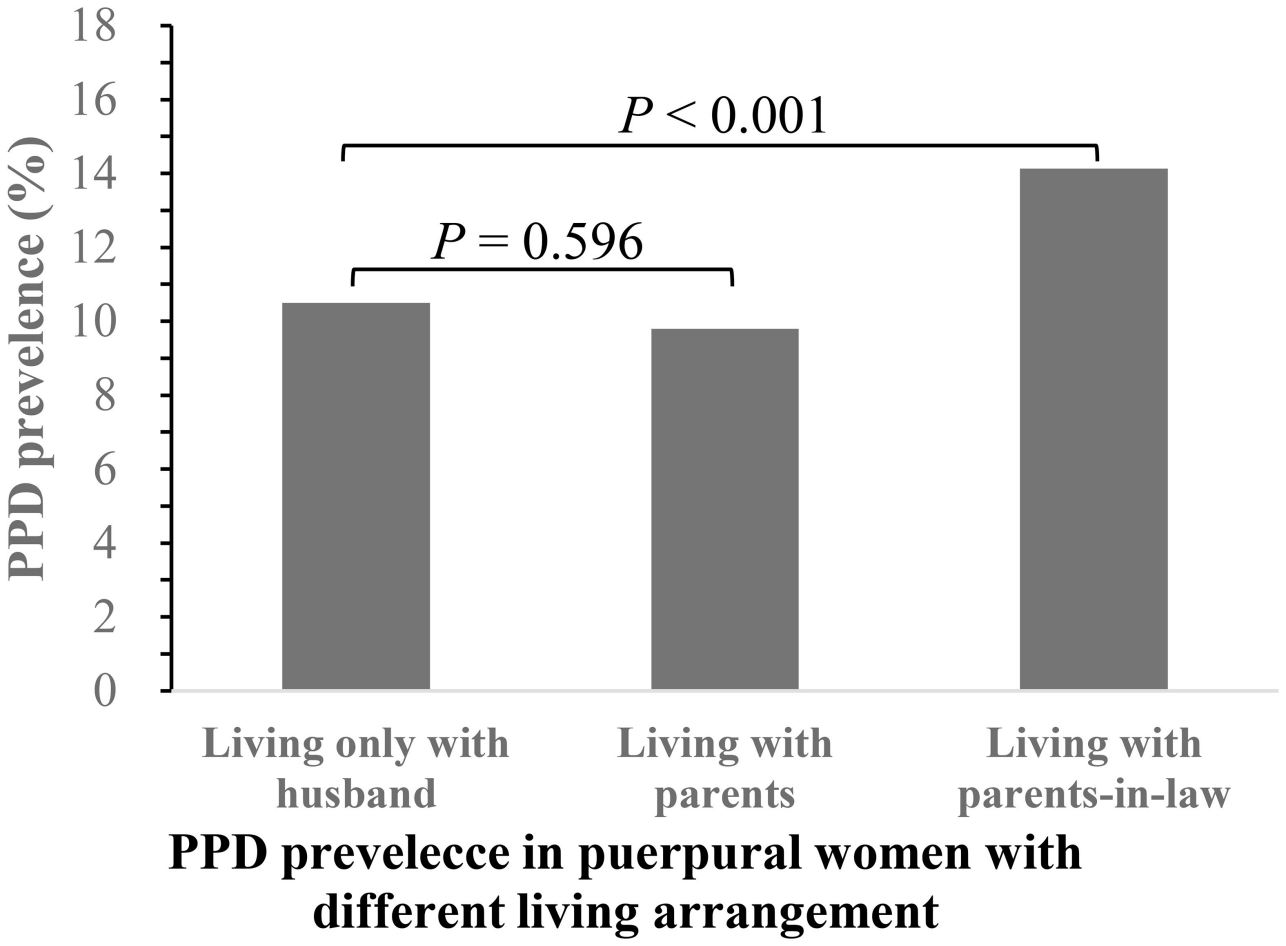
The PPD prevalence in puerpural women with different living arrangement.

Of the 4,813 puerperal women enrolled in this study, 559 (11.6%) were considered to have PPD. The average age of the participants was 29 years, with an interquartile range from 27 to 32 years old. According to the puerperal women's living arrangements, we divided these women into three groups: (1) living only with their husband; (2) living with their parents; and (3) living with their parents-in-law. The numbers of women in these three groups were 2,535 (52.7%), 664 (13.8%), and 1,614 (33.5%), respectively, and the corresponding prevalence of PPD was 10.5, 9.8, and 14.1%, respectively (shown in Figure 1). The prevalence of PPD was significantly different between women living only with husbands and women living with parents-in-law (*P* < 0.001). However, there was no significant difference in the prevalence of PPD between women living with parents and women living only with husbands (*P* > 0.05).

Table 1 shows the demographic characteristics and pregnancy-related factors of the three groups. Women living with their parents-in-law were younger and had lower rates of primipara and cesarean section than women in the other two groups. In addition, there were significant differences in education level, employment status, and breastfeeding status at 6 weeks among the three groups. However, other demographic characteristics or pregnancy-related factors did not show significant differences among the groups, in terms of past psychiatric history, family history of mental illness, GDM, PIH, depression, and anxiety during pregnancy, stressful life events, male infant, preterm birth, low birth weight, and malformation.

**Table 1:** Social-demographic characteristics according to PPD status of women.

**Characteristics**	**Living only with husband**	**Living with parents**	**Living with parents-in-law**	***P*-value**
	**(*n* = 2,535)**	**(*n* = 664)**	**(*n* = 1,614)**	
Age at birth (years)	29.78 ± 4.29	29.88 ± 4.30	28.65 ± 4.06	<0.001
Education age	13.61 ±2.70	14.16 ± 2.56	14.01 ± 2.51	<0.001
Education level				<0.001
Junior middle school or less	313 (12.3%)	62 (9.3%)	154 (9.5%)	
Senior middle school	782 (30.8%)	161 (24.2%)	426 (26.4%)	
College or university	1,440 (56.8%)	441 (66.4%)	1,034 (64.1%)	
Employment status				0.001
Full-time employed	1,437 (56.7%)	385 (58.0%)	976 (60.5%)	
Self-employed	399 (15.7%)	111 (16.7%)	196 (12.1%)	
Housewife	263 (10.4%)	47 (7.1%)	140 (8.7%)	
Other	436 (17.2%)	121 (18.2%)	302 (18.2%)	
Past psychiatric history	24 (0.9%)	7 (1.1%)	7 (0.4%)	0.135
Family history of mental illness	37 (1.5%)	5 (0.8%)	27 (1.7%)	0.242
Primipara	1,167 (46.0%)	292 (44.0%)	637 (39.5%)	<0.001
Gestational diabetes mellitus	185 (7.3%)	44 (6.6%)	117 (7.2%)	0.832
Pregnancy-induced hypertension	105 (4.1%)	20 (3.0%)	47 (2.9%)	0.081
Depression during pregnancy	178 (7.0%)	46 (6.9%)	135 (8.4%)	0.236
Anxiety during pregnancy	420 (16.6%)	107 (16.1%)	284 (17.6%)	0.594
Stressful life events	97 (3.8%)	28 (4.2%)	72 (4.5%)	0.594
Male infant	1,342 (52.9%)	352 (53.0%)	854 (52.9%)	0.999
Cesarean delivery	884 (34.9%)	232 (34.9%)	489 (30.3%)	0.006
Gestational weeks	38.87 ± 1.59	38.79 ± 1.82	38.88 ± 1.63	0.456
Preterm birth	145 (5.7%)	43 (6.5%)	86 (5.3%)	0.560
Birth weight	3219.02 ± 467.26	3215.21 ± 492.76	3208.20 ± 464.89	0.770
Low birth weight	125 (4.9%)	38 (5.7%)	78 (4.8%)	0.655
Malformation	56 (2.2%)	10 (1.5%)	31 (1.9%)	0.490
Breastfeeding status at 6 weeks				<0.001
Exclusive	1,640 (64.7%)	368 (55.4%)	938 (58.1%)	
Partial	773 (30.5%)	252 (38.0%)	571 (35.4%)	
Formula only	122 (4.8%)	44 (6.6%)	105 (6.5%)	

#### Living Arrangement and PPD Risk

The results of the unadjusted and adjusted logistic regression analyses are listed in Table 2. We used four logistic regression models to control for the confounders of PPD. Model 1 (the unadjusted model) showed that women who lived with their parents-in-law (OR = 1.40; 95% CI, 1.16–1.70) had a higher risk of developing PPD than women who lived only with their husbands. Model 2 indicated that women living with their parents-in-law (OR = 1.38; 95% CI, 1.14–1.67) had an increased risk of developing PPD after adjusting for age, education, employment status, past, and family history of mental illness. In previous studies (33, 34), these covariates have been reported to be associated with the PPD risk. In Model 3, we adjusted for the effects of parity, GDM, PIH, depression and anxiety during pregnancy, and stressful life events on outcome variable, and we observed that women who lived with their parents-in-law had a significantly higher risk of developing PPD (OR = 1.40; 95% CI, 1.14–1.72) than those who only lived with their husbands. Finally, in Model 4, we made additional adjustments for the infant sex, mode of delivery, preterm birth, low birth weight, malformation, and feeding pattern at 6 weeks, which were also risk factors for PPD. The ORs remained statistically significant, with a value of 1.38 (95% CI, 1.12–1.70) for women who lived with parents-in-law compared with women who only lived with their husbands. However, in the 4 models, there was no significant difference in the risk of PPD between women who lived with their parents and those who lived only with husbands.

**Table 2:** OR (95% CI) of postpartum depression according to puerperal women's living situation.

**Characteristics**	**Living only with husband**	**Living with parents**	***P*-value**	**Living with parents-in-law**	***P*-value**
No. of participants	2,535	664	–	1,614	–
Model 1	Ref.	0.93 (0.70–1.23)	0.596	1.40 (1.16–1.70)	<0.001
Model 2	Ref.	0.96 (0.72–1.28)	0.763	1.38 (1.14–1.67)	0.001
Model 3	Ref.	0.97 (0.72–1.32)	0.868	1.40 (1.14–1.72)	0.001
Model 4	Ref.	0.95 (0.70–1.29)	0.739	1.38 (1.12–1.70)	0.002

*OR, odds ratio; CI, confidence interval*.

*Model 1: unadjusted*.

*Model 2: adjusted for age, education level, employment status, past psychiatric history, family history of mental illness*.

*Model 3: adjusted for covariates in model 2 and parity, GDM, PIH, depression during pregnancy, anxiety during pregnancy, stressful life events*.

*Model 4: adjusted for covariates in model 3 and infant gender, mode of delivery, preterm birth, low birth weight, malformation, feeding method at 6 weeks*.

### Interaction of Other Variables and Living Arrangement

**Figure 2:**
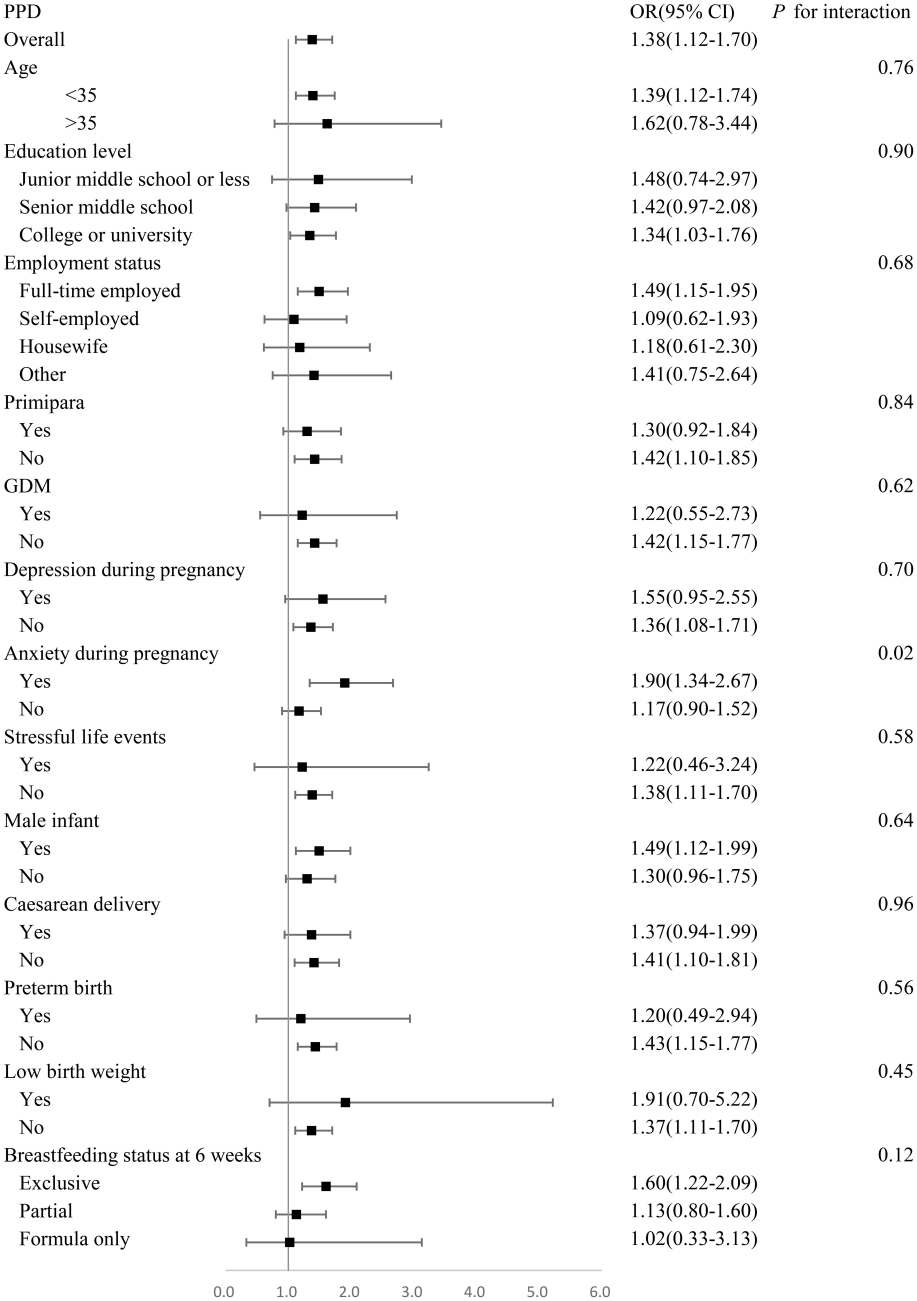
Adjusted odds ratios for prevalence of PPD stratified by age, education, employment, parity, GDM, depression during pregnancy, anxiety during pregnancy, stressful life events, infant gender, mode of delivery, preterm birth, low birth weight, feeding method at 6 weeks. Analyses are adjusted for age, education, employment, past psychiatric history, family history of mental illness, parity, GDM, PIH, depression during pregnancy, anxiety during pregnancy, stressful life events, infant gender, mode of delivery, preterm birth, low birth weight, malformation, feeding method at 6 weeks.

Stratification analyses revealed that the association between living with parents-in-law and the presence of PPD was more significant in women with anxiety during pregnancy (*P* for interaction <0.05). No interaction was observed between livingwith parents-in-law and the presence of PPD in women with any other variables (*P* for interaction >0.05) (Figure 2).

## Discussion

This study showed that women living with their parents-in-law were younger, and had fewer childbirth experiences, lower cesarean section rate, a higher education level, employment rate, and formula feeding rate. In addition, our study suggested that maternal living arrangements were associated with the risk of PPD. Women who lived with their parents-in-law had an increased risk of developing PPD, independent of other PPD risk factors. However, these women living with their parents did not show an increased risk of PPD.

Our study showed that living with parents-in-law was associated with a higher risk of PPD in puerperal women, which is consistent with previous studies (20, 25). The preliminary investigation conducted by Wang et al. indicated that mothers living with their parents-in-law had an additional risk for PPD in the Chinese population, with an OR value of 2.48 (95% CI: 1.20, 5.15) (20). Additionally, a large-scale cohort study in Japan found that puerperal women who did not live with their parents-in-law had a lower risk of PPD compared with those who lived with these family members (25), suggesting that co-residency with parents-in-law may damage postpartum mental status. However, a study in Nepal focused on similar areas reported that postpartum depression was associated with living in nuclear families, and puerperal women living in nuclear families had an increased risk of PPD than women living in extended families (OR 48.5) (35). However, the reliability of their results is limited due to the small sample size.

Previous surveys have shown that disharmony between mothers-in-law and daughters-in-law increases the risk of PPD among Chinese women (32, 36). In China, the contradiction between mothers-in-law and daughters-in-law is very common, especially those who live together. In the traditional concept, daughters-in-law should respect their elders and obey the wills of the elders. However, the new generation of women have received more education and have different thoughts and concepts. For those who take care of their children, new mothers may stand up for what they believe is right, which may be against the wills of their elders. Traditional concepts are challenged by modern thinking patterns, which leads to intense in-law conflicts within the families, partly explaining why living with parents-in-law increases the risk of PPD risk in puerperal women.

We observed an interaction between living with parents-in-law and anxiety during pregnancy in the presence of PPD. The relationship between living with their parents-in-law and the presence of PPD was more obvious among anxious mothers. To the best of our knowledge, no evidence is available for the interaction between living with parents-in-law and anxiety during pregnancy on the risk of PPD. However, it has been noticed that anxiety during pregnancy is associated with a poor marital relationship (37), which is identified to be a typical risk factor for PPD. Additionally, anxiety can easily lead to tension and irritability (38), which can lead to bad interpersonal relationships among women. Therefore, we speculate that women who experience anxiety during pregnancy are more likely to have poor family relationships, including marital relationships and in-law relationships, resulting in a higher risk of PPD for women who live with their parents-in-law.

## Limitations

There is no doubt that this study has several limitations. First, our study was cross-sectional in design, and we could not infer the causal relationship between living with parents-in-law and the risk of PPD. Therefore, a cohort study is needed to verify this causal relationship. Second, we did not collect data on the relationship between mothers-in-law and daughters-in-law, which prevents us from exploring whether living with parents-in-law affects the risk of PPD in puerperal women because of an unsatisfactory relationship between mothers-in-law and daughters-in-law. Third, EPDS is only a tool for screening PPD, not a diagnostic tool. Further diagnosis requires the professional judgment by a psychiatrist.

## Conclusions

In summary, our study demonstrated that living with their parents-in-law was associated with the risk of PPD among Chinese puerperal women. Furthermore, anxiety during pregnancy may mediate the relationship between living with parents-in-law and PPD. The effect of maternal living arrangements on PPD should be taken into consideration to prevent PPD. Further studies are needed to explore specific mechanisms underlying this association.

## Data Availability Statement

The raw data supporting the conclusions of this article will be made available by the authors, without undue reservation.

## Ethics Statement

The studies involving human participants were reviewed and approved by Institutional Review Committee of Baoan Maternity and Child Health Hospital. The patients/participants provided their written informed consent to participate in this study.

## Author Contributions

SP did the statistical analysis and drafted the initial manuscript. XL, JQ, and YD contributed to assist with data collection and revised the manuscript. JY, YB, and YJ took part in the sample collection. LM, KW, and XZ contributed to the critical revision of the article. All authors contributed significantly to this work and have approved the final manuscript.

## Conflict of Interest

The authors declare that the research was conducted in the absence of any commercial or financial relationships that could be construed as a potential conflict of interest.

## Publisher's Note

All claims expressed in this article are solely those of the authors and do not necessarily represent those of their affiliated organizations, or those of the publisher, the editors and the reviewers. Any product that may be evaluated in this article, or claim that may be made by its manufacturer, is not guaranteed or endorsed by the publisher.
